# Measurement of the visual axis through two different methods: quantification and differences for measuring chord µ

**DOI:** 10.5935/0004-2749.2022-0035

**Published:** 2023-03-08

**Authors:** Pablo Felipe Rodrigues, Bernardo Kaplan Moscovici, Luciano Lamazales, Marcela Mara Silva Freitas, José Álvaro Pereira Gomes, Walton Nosé, Mauro Silveira Campos

**Affiliations:** 1 Department of Ophthalmology and Visual Sciences, Escola Paulista de Medicina, Universidade Federal de São Paulo, São Paulo, SP, Brazil; 2 Cornea and Diseases Sector of the Ocular Surface, Instituto Suel Abujamra, São Paulo, SP, Brazil

**Keywords:** Optical imaging, Visual perception, Pupil, Anterior eye segment, Cornea, Diagnostic techniques, ophthalmological, Imagem óptica, Percepção visual, Pupila, Segmento anterior do olho, Córnea, Técnicas de diagnóstico oftalmológico

## Abstract

**Purpose:**

To compare the differences between the *apparent* and *actual chord* µ.

**Methods:**

In this prospective, comparative, non-randomized, and non-interventional study, imaging examinations using Pentacam and the HD Analyzer were performed in the same room under the same scotopic conditions. The inclusion criteria were patients aged 21-71 years, able to provide informed consent, myopia up to 4D, and anterior topographic astigmatism up to 1D. Patients using contact lenses, those with previous eye diseases or surgeries, corneal opacities, corneal tomographic changes, or suspected keratoconus were excluded.

**Results:**

Altogether, 116 eyes of 58 patients were analyzed. The patients’ mean age was 30.69 (±7.85) years. In the correlation analyses, Pearson’s correlation coefficient of 0.647 indicates a moderate positive linear relationship between *apparent* and *actual chord* µ. The mean *actual* and *apparent chord* µ were 226.21 ± 128.53 and 278.66 ± 123.90 µm, respectively, with a mean difference of 52.45 µm (p=0.01). The analysis of mean pupillary diameter resulted in 5.76 mm using the HD Analyzer and 3.31 mm using the Pentacam.

**Conclusions:**

We found a correlation between the two measurement devices, and even though we found considerable differences, both can be used in daily practice. Given their differences, we should respect their peculiarities as well.

## Abstract (pt)

**Objetivo:**

Comparar as diferenças entre a *chord* aparente µ e o *chord* real µ.

**Métodos:**

Estudo prospectivo, comparativo, não randomizado e não intervencionista. Os exames de imagem (Pentacam e HD Analyzer) foram realizados na mesma sala e nas mesmas condições escotópicas. Os critérios de inclusão foram idade de 21 a 71 anos; compreensão do termo de consentimento; miopia até 4D e astigmatismo topográfico anterior até 1D. Os critérios de exclusão foram usuários de lentes de contato; pacientes com doenças oculares prévias ou cirurgias; opacidades da córnea; a presença de alterações tomográficas da córnea ou suspeita de ceratocone.

**Resultados:**

Em nosso estudo foram analisados 116 olhos de 58 pacientes. A média de idade foi de 30,69 anos (± 7,85). Análises de correlação foram desenvolvidas e o coeficiente de correlação de Pearson (0,647) indica uma relação linear positiva moderada entre as variáveis. A média do *chord* µ real foi 226,21 ± 128,53 µm e a média do *chord* µ média foi 278,66 ± 123,90 µm, com diferença média de 52,45 µm (p=0,01). A análise do diâmetro pupilar médio apresentou: 5,76mm no HD Analyzer e 3,31mm no Pentacam.

**Conclusões:**

Entendemos a existência de uma diferença significativa entre os métodos e assim a medida de ambos os dispositivos com base em princípios diferentes devemos respeitar suas peculiaridades. Como encontramos correlação entre as duas medidas, acreditamos que ambas podem ser utilizadas na prática diária.

## INTRODUCTION

A perfect optical model would be represented by an imaginary line between the fixation object and optical centers of all the ocular elements directly to the foveol^(1)^. The human eye is not a perfect optical system^(2)^. This is true because the reference to nodal points persist metaphorically. Thus, they represent mathematical constructions rather than anatomical references^(3)^. Therefore, the objective of creating a visual axis between the fixation object and fovea is attributed to the nodal points.

Contrarily, there is a divergence between the corneal reference to be adopted to obtain the best eye centering for glasses, contact lenses, and even refractive surgeries^(2)^. Recently, the relevance of adopting the distance between the center of the entrance pupil and the corneal reflex (caused by an object fixed coaxially to the eye) has been widely discussed, as has the meaning of the distance between these reference points for ocular centralization. The Purkinje reflex or Purkinje-Sanson is the reflection of objects in the eye structure that can form four different images. Thus, the Purkinje reflection will usually not be centered when fixing a point of light. The most important Purkinje reflex is the first image, which is the reflection on the outermost surface of the cornea (the closest point to the placid disc in a topography, for example). After the light source is reflected, four Purkinje images (P1, P2, P3, and P4) are formed, although only three (P1, P3, and P4) are appreciated clinically. In this study, P1 refers to the Purkinje reflex^(1^-^6)^.

The kappa angle (originally the lambda angle) is the angle between the visual and pupillary axes. The pupillary axis is a line perpendicular to the cornea that passes through the center of the pupil. The visual axis is the line connecting the fixation point with the foveola, passing through the two nodal points of the eye. It can be determined by locating the reflected image of the light source (viewed from the source) in the cornea (first Purkinje image). Recently, a more appropriate term, chord length µ (µm), has been suggested. Chord length µ denotes the two-dimensional displacement of the pupillary center from the subject-fixated coaxially sighted corneal light reflex that references the distance between two points, rather than the angle^(7^-^10)^.

**Figure 1:**
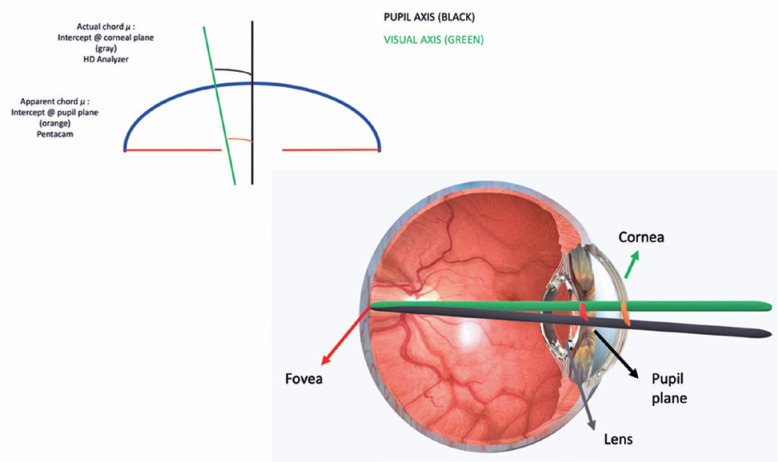
Aschematic representation of the study concepts.

*Chord* µ represents the displacement of the pupillary center of entry of the coaxially sighted corneal light reflex. The *apparent chord* µ is the distance between the Purkinje reflex and the apparent pupil center when viewed coaxially from the light source at the cornea. In contrast, the *actual chord* µ is the actual distance between the visual axis and pupil center (at the pupil plane), which is lesser because it is not magnified by the cornea (Figure 1). Although the actual *chord*µ refers to the distance between two points on a given plane rather than the angles between two lines, it changes as the frame of reference moves from the iris lens plane to the corneal plane^(11^-^14)^.

Device measurement requires a high-contrast refe-rence that can be continuously recognized by an infrared camera or visible light, either during surgeries or examinations. The color difference between the iris and pupil generates a sufficient contrast for such a mechanism^(7^-^9)^. Therefore, more-accurate ocular reference marker measurements corresponding with better centralization for refractive procedures and, consequently, better visual quality.

This study aimed to quantify the measurements of *chord* µ obtained by two different devices, i.e., HD Analyzer (Visiometrics, Cerdanyola del Vallès, Halma Company™) that measures the *apparent chord* µ and Pentacam HR, which measures the *actual chord* µ (Oculus, Wetzlar, optikgeräte GmbH) and to determine the difference between them.

## METHODS

This prospective, comparative, non-randomized, and non-interventional study complied with the standards stipulated in the Declaration of Helsinki and was approved by the Research Ethics Committee of Suel Abumjara (36907320.9.0000.5477). Informed consent was obtained from the patients before study participation. All examinations were performed in a private clinic (Clinic Spot, São Paulo, Brazil). The same professional performed the imaging examinations before the other clinical examinations, in a random order, in the same room under the same scotopic conditions.

The inclusion criteria were as follows: aged 21-71 years; able to provide consent; with myopia up to 4D; and with anterior topographic astigmatism up to 1D. We excluded patients who used contact lenses, those with previous eye diseases or surgeries, those with corneal opacities, those with corneal tomographic changes, and those with suspected keratoconus.

### Acquisition of images for both devices

The patient’s chin was properly supported for both tests, and the patient’s forehead was pressed against the specific strip. A central fixation light aligned the eye with the visual axis. The examiner saw a real-time image of the eye on the screen. We obtained five acquisitions per eye. When the image is focused and centered, the software starts the measurements automatically. The patient was asked to remain still with eyes open. The same trained operator performed the examinations. Both devices (Pentacam and HD Analyzer) have test reliability indexes, and in case of unreliable tests, the test was repeated until the measurements were considered reliable.

### Apparent and actual chord u

The HD Analyzer results were provided after five measurements, validated by the device’s software (and the system automatically chooses the more reliable measurement to be adopted). The display shows the geographical position in a two-dimensional plane (X and Y axes) and *apparent chord* µ from the pupillary center to the Purkinje reflex. Previous studies have already demonstrated the reproducibility and reliability of this measurement method^(10^-^12)^.

The Pentacam™ uses the Purkinje reflex as the primary reference. The camera reports the distance from the pupil center to the visual axis (*actual chord* µ), which is considered the center of the x and y coordinates. A negative x-axis value in the right eye and a positive value in the left eye indicate that the pupil is temporal to the light reflex. Similarly, negative values along the y-axis denote an inferior pupil center location. The display maintains the cartographic orientation (the right has positive and left negative signs) independent of the evaluated eye. The tests considered suitable for this study adhered to the manufacturer’s reliability specifications^(12^,^13)^.

**Figure 2:**
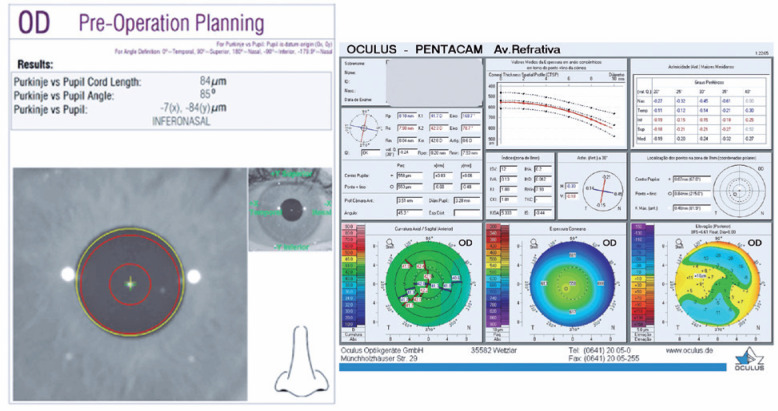
Two tests using the HD Analyzer and Pentacam HR are performed on the same eye of the same patient. Left: examination with the HD Analyzer. Right; examination with the Pentacam HR.

Figure 2 shows the two devices and their supposed cartographic representations of the *chord* µ.

### Statistical analysis

Descriptive, correlation, *t*-tests, and Bland-Altman tests were performed; We analyzed the data in Stata^®^17^(12^-^14)^.

## RESULTS

In our study, we analyzed 116 eyes of 58 patients, including 29 males and 29 females. The patients’ mean age was 30.69 (±7.85) years. The descriptive results are shown in Table 1.

**Table 1:** Descriptive analysis of Pentacam and HD hypotenuse (actual and apparent chord µ)

		Actual chord µ	Apparent chord µ
		Sample	Sample
N		116	116
Mean		226.21	278.66
Median		215.00	266.50
Std. deviation		128.53	123.90
Minimum		10.00	24.00
Maximum		600.00	633.00
Percentiles	25	122.50	202.75
	50	215.00	266.50
	75	290.00	338.25

The mean *actual* and *apparent chord* µ were 226.21 ± 128.53 and 278.66 ± 123.90 µm, respectively, with a mean difference of 52.45 µm (p=0.01).

A correlation analysis was performed to determine the relationship between the Pentacam and HD Analyzer variables. The Pearson’s correlation coefficient of 0.647 indicates a moderate positive linear relationship between the variables of the Pentacam (*actual chord* µ) and HD Analyzer (*apparent chord* µ) with a p-value (<0.001); in other words, when the *chord*µ values are high, they were high on both devices. The same interpretation could be made when both values were low.

**Figure 3:**
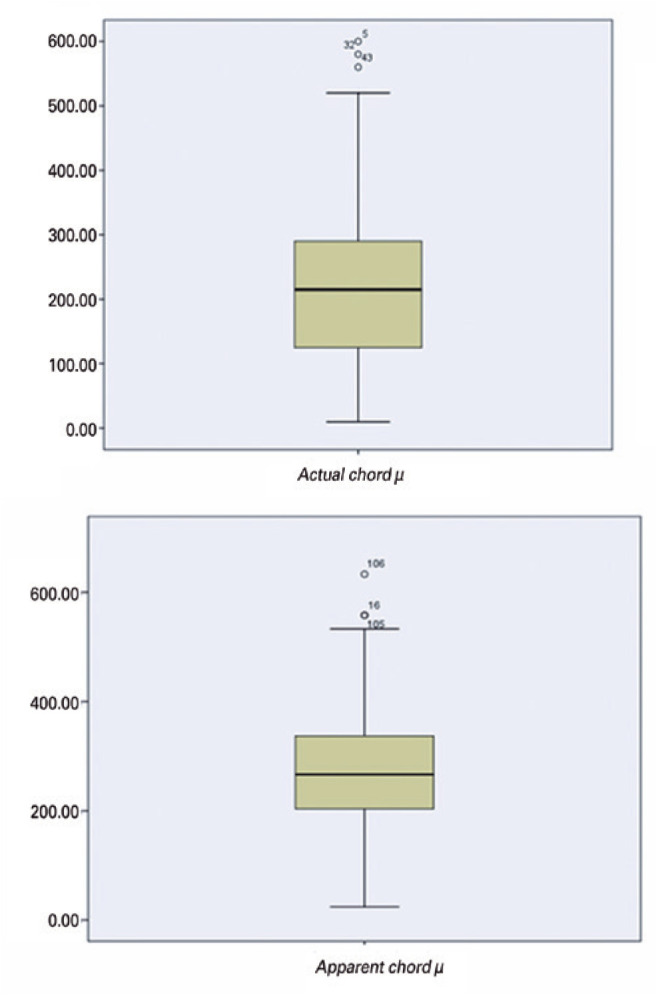
Boxplot of the hypotenuse between the Pentacam HR and HD Analyzer (*actual* and *apparent chord* µ).

An agreement analysis was carried out between the two methods (Bland-Altman). The mean difference between the two methods was 52.45 µm with a standard deviation (SD) of 106.17 µm. The upper and lower 95% confidence interval (CII) limits were 260.55, and -155.64, respectively. The one-sample *t*-test with the differences shows a *t*-statistic of 5.3 and a p-value of 0.00, which means the null hypothesis that the differences are equal to zero is rejected. Hence, the results show that the two devices did not agree with their hypotenuse measurements (Figure 3). Figure 5 presents the Bland-Altman graph^(15)^.

**Figure 4:**
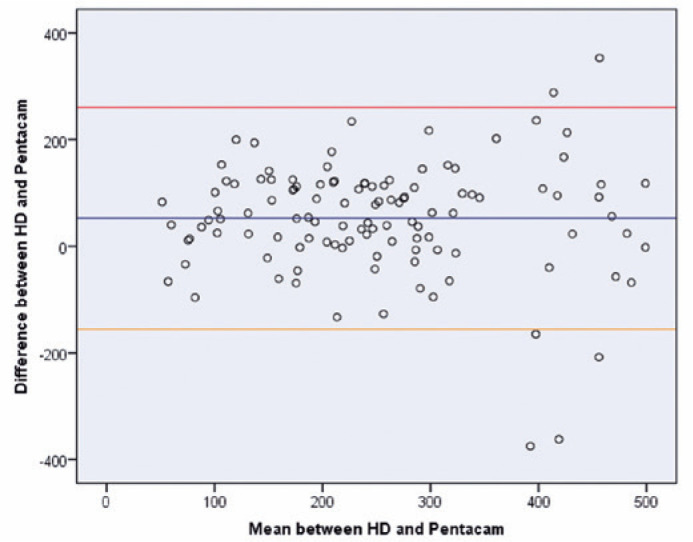
Bland-Altman graph of the hypotenuse. The blue lines refer to the mean (52.46 µm), the red lines refer to the upper range (260.55 µm), and the orange lines refer to the lower range (-155.64 µm).

**Figure 5:**
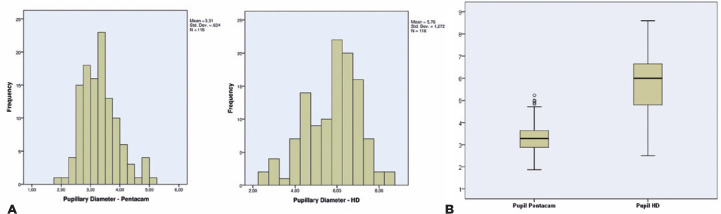
(A). Presentation of the pupillary diameter distribution between the two devices. (B) Boxplot of the pupillary diameter obtained by the two devices (Pentacam HR and HD Analyzer).

In the correlation analysis between the pupillary dia-meter and measurement corresponding to each nodal point of the visual axis, the HD Analyzer showed an average pupil diameter of 5.76 mm (2.50-8.60 mm). In contrast, Pentacam demonstrated an average of 3.31 mm (1.87-5.23 mm). In the HD Analyzer sample, 75% had a lower pupillary diameter of 6.67 mm, while in the Pentacam sample, 75% had a lower pupil diameter of 3.63 mm, as shown in figure 4.

## DISCUSSION

We are faced with two realities and two needs. One is the device’s capacity and technical requirements; here, contrast areas must function appropriately. In contrast, the human optical system (despite recognizing the most prominent light source in the entrance pupil) does not necessarily have a central alignment between the fixation object and macula. Thus, the correct measurement of the *chord* µ is directly associated with the success of refractive surgeries and intraocular lens choices, especially the diffractive ones^(11)^.

Holladay et al. reported that the mean *apparent chord* µ is 0.3 ± 0.15 mm, with an upper limit of normal of 0.60 mm (mean ± 2.0 *SD*). The value of the *actual chord* µ was 0.2 ± 0.11 mm; thus, the upper limit of normal values at a 95% CI for the *actual chord* µ would be 0.42 mm^(11)^. As expected, we found greater values in the *apparent chord* µ, similar to Holladay’s description, but the difference between the measurements was smaller in our study.

The presence of a correlation demonstrates that measurements obtained by both devices were positively related; however, the Bland-Altman test shows that these devices do not agree with their measurements^(16^-^18)^. However, slightly different measurements are inevitable as the devices measure two different variables. It is more important to determine the amount by which these measurements disagree. Our study results showed that these two devices present an acceptable relationship; thus, both devices can be used in clinical practice.

Setting a limit on the longest distance between measurements will depend on how the results will be used; thus, it is a matter of clinical judgment. A mean difference of 86.75 µm was found between the two devices in the same patients. The *chord* µ *(actual)* mea-surement, obtained by Pentacam, is performed with visible blue spectrum light, causing a more significant pupillary constriction than the HD Analyzer (*apparent*), which uses infrared light. This factor can influence the actual *chord* µ measurement. A 0.20 ± 0.11 mm CI for Pentacam suggests a better correspondence to the HD Analyzer (double-pass technique)^(11)^.

Even a slight difference can influence some aspects of clinical practice, such as the choice of diffractive intraocular lenses. Diffractive intraocular lenses can be contraindicated in patients with high *chord* µ values. If there is a difference in the measurements between the devices, their indication may be compromised in these cases. The same can be said for refractive surgeries and corneal inlays dependent on the *chord* µ values.

However, adopting ocular interdependence to accept binocular evaluation may be questioned, despite the sample obtained. Although no cases had previous surgeries and posterior face changes, correlation changes may be associated with an increase in the sample.

The dependence on both methods is associated with adequate optical understanding directly related to the fixation capacity for image capture. Therefore, despite the scientific and technological advances in measuring ocular architecture, medical criteria for treating patients, rather than examinations, are still necessary.

Achieving better ocular centralization in refractive surgeries and multifocal intraocular lens implants should translate into surgical successes. This feature has been suggested in other studies, especially in patients with increased mu or kappa angles. Unfortunately, more-pre-cise devices, like the HD Analyzer, are more difficult to find in daily practice, unlike Scheimpflug Tomography devices, such as the Pentacam. Therefore, the correlation between µ chord measurements suggests the relevance of the measurement of the visual axis. Application of the real chord u may be useful for ophthalmologists making preoperative decisions^(12)^.

In conclusion, we now better understand the relationship between these two measurement methods; thus, each device’s peculiarities should be considered based on their different principles. Since the measurements by the two devices were correlated, despite noting considerable differences, we suggest that both tools can be used in daily practice.
